# Utilization of the Intimacy and Sexuality Expression Preference tool in long-term care: a case study

**DOI:** 10.3389/frdem.2024.1270569

**Published:** 2024-02-26

**Authors:** Cindy Jones, Wendy Moyle, Kimberly Van Haitsma, Carly Hudson

**Affiliations:** ^1^Faculty of Health Sciences and Medicine, Bond University, Gold Coast, QLD, Australia; ^2^Menzies Health Institute Queensland, Nathan, QLD, Australia; ^3^School of Nursing and Midwifery, Griffith University, Nathan, QLD, Australia; ^4^Ross and Carol Nese College of Nursing, Pennsylvania State University, University Park, PA, United States

**Keywords:** intimacy, sexuality, sexual wellness, aged care, dementia, preference tool

## Abstract

**Introduction:**

Sexual wellness plays a crucial role in an individual's quality of life, interpersonal relationships, and self-concept, particularly among older adults residing in residential aged care facilities, including those with dementia. However, there is currently a limited person-centered approach to understanding the unique preferences of each older person regarding their intimate and sexual behaviors. To address this gap, the *Intimacy and Sexuality Expression Preference* (ISEP) tool was developed to facilitate meaningful discussions between healthcare professionals or workers and older individuals about their intimacy and sexuality needs and preferences. This paper explores the use of the ISEP tool with residents in long-term aged care, including those with dementia via a user-centric case study.

**Methods:**

ISEP tool interviews were conducted with 14 residents in a single residential aged care facility in Queensland, Australia.

**Results:**

The study presented valuable insights and contextual information from using the ISEP tool, including an example of a resident's response, which provided recommendations for better supporting the resident. This involves engaging in supportive conversations to facilitate the exploration, implementation, and assessment of practical and actionable strategies to meet intimacy and sexuality needs and preferences.

**Discussion:**

The ISEP tool shows promise in improving care practices and addressing the intimacy and sexuality needs of older individuals in aged care facilities. However, it is important to acknowledge that the study was conducted in a single aged care facility with a small group of residents, potentially limiting the generalisability of the findings. Further large-scale studies are necessary to establish the tool's broader applicability across different care settings.

## 1 Introduction

The World Health Organization (WHO) defines sexual health as “*a state of physical, emotional, mental, and social wellbeing in relation to sexuality”* (World Health Organization, 2006). Healthcare professionals (i.e., trained and qualified to provide healthcare services for example, doctors, nurses and allied health practitioners) widely recognize that supporting older people, including those living with dementia, to express intimacy and sexuality is an important aspect of holistic care in older people (Haboubi and Lincoln, 2003; Byrne et al., 2010; DeLamater, 2012; Fennell and Grant, 2019). Dementia profoundly impacts the life of the older person and their family, extending to their intimate relationships and the expression of sexuality. Broaching the topic of sexual needs and preferences amongst older people can pose challenges due to its sensitive nature. Nevertheless, older people, including those living with dementia, can form new and meaningful intimate relationships, and may express a desire to engage in purposive sexual and intimate behaviors (Everett, 2007). Whilst sexual health is known to be an important aspect of care for older people, it is often neglected (Jones and Moyle, 2018).

Sexual wellness is a key aspect of health that contributes significantly to an individual's quality of life, maintenance of interpersonal relationships and positive self-concept (Robinson and Molzahn, 2007). Sexual expressions by older people living in residential aged care facilities (RACFs) can include a range of activities, such as maintenance of physical appearance, physical contact and displays of affection, flirting, engaging with sexually explicit materials, masturbation, or sexual intercourse with current or new partners, whether they reside inside or outside RACFs (Jones et al., 2021). Whilst these are all positive behaviors, feelings of uneasiness and discomfort can result for healthcare professionals who encounter them. In older people living with dementia, these behaviors are often considered inappropriate, or their validity is questioned. This can occur because health professionals may interpret these behaviors as responsive symptoms of dementia, leading to discouragement and attempts to suppress them (Jones et al., 2021). Despite their sensitive nature, older people desire the opportunity to discuss their sexual health concerns. This is reinforced by health professionals who feel that sexual wellness should be included in holistic care (Haboubi and Lincoln, 2003). To support the sexual health and wellbeing of older people, particularly those living with dementia in RACFs, sexual needs and preferences must be respected (World Health Organization, 2006). Holistic care is a personalized approach to care, emphasizing the need to “*know the person”* (Gannod et al., 2019). Delivery of person-centered care can support older people's sexual health and wellbeing by assisting to reduce adverse behaviors, which are displayed when sexual feelings, desires, and needs are not being met (Jones et al., 2021). Therefore, it is essential to understand the preferences of each older person to express their preferred intimate and sexual behaviors meaningfully.

### 1.1 Intimacy and Sexuality Expression Preference tool

The *Intimacy and Sexuality Expression Preference* (ISEP) tool was developed to enhance discussion between a healthcare professional or worker (e.g., RACF manager) and an older person regarding their intimacy and sexuality needs and preferences. The tool, in the format of a questionnaire form, also supports the older person in meeting their identified needs (Jones et al., 2021). The ISEP tool was created using the Delphi technique to gain consensus and expertise across a panel of individuals interested in or knowledgeable in this field. The ISEP tool explores an older person's preferences across 10 content areas: preferred name and pronunciation, gender identity, intimacy, sexuality, sex, sexual orientation, romantic and/or sexual relationships, safe sex, other aspects such as history, abuse or trauma, and supporting intimacy and sexual needs and preferences.

To achieve personalized holistic care, understanding the older person is required. By collecting this information, the ISEP tool can potentially enable the provision of person-centered care. Assessing intimacy and sexuality preferences is essential for person-centered care delivery to promote health and wellbeing, prevent adverse behaviors due to unmet needs and address concerns when the appropriateness of expressions of intimacy and sexuality are raised. It can facilitate the collaborative work between the healthcare team, the older person and their family to address their identified needs and preferences (Jones et al., 2021). The development process of the ISEP tool, including content validation, and a downloadable copy of it can be accessed through the published peer-reviewed article (Jones et al., 2021).

This study examines the utilization of the ISEP tool within a long-term aged care environment, with a specific focus on older individuals, including those affected by dementia. A user-centric case study is presented to explore how the ISEP tool may inform care practices in the long-term aged care setting. This paper provides insights for healthcare professionals or workers, showcasing how the tool can be used to improve care quality for older individuals, including those with dementia. It offers practical advice for incorporating the tool into care routines, ultimately enhancing long-term aged care.

## 2 Design and methods

### 2.1 Case study design

This study received approval from the Bond University Human Research Ethics Committee #CJ00282. A feasibility study design involving multi-phase mixed methods was originally planned that included a one-day educational training workshop for care staff. This was then followed by data collection involving completing various surveys and questionnaires by participants (i.e., facility managers, care staff, older people and their families pre- and post-ISEP tool implementation). Accredited RACFs in Queensland, Australia, were contacted with information about the study's purpose and the nature of participation, along with a copy of the ISEP Tool for reference. They were requested to contact the research team to express their interest in participating. However, the study experienced significant disruptions due to the coronavirus 2019 pandemic that impacted the ability to carry out planned activities, plus as a result, a general reluctance among aged care organizations to participate in research activities during and after the pandemic's peak. Despite the initial interest and consent from several RACFs to participate in the study, only one RACF ultimately engaged in the research due to the pandemic-related constraints. Consequently, the ISEP tool was utilized and evaluated within a case study framework, which focused on a single RACF and included interviews with its residents, as outlined in Section 2.2.

### 2.2 ISEP interview—RACF residents and procedure

The case study presented in this paper was carried out in an Australian north Queensland RACF where residents (i.e., older people) were invited to take part in an ISEP interview. The facility manager distributed the study information sheet to residents living with and without early to mid-stage dementia. Permission was requested from prospective residents and their legally authorized representatives (LAR), including family members, partners/spouses, and legally appointed guardians where necessary and applicable. This permission was requested to initiate a discussion with them regarding their potential involvement in an interview to answer the questions in the ISEP tool. The researcher then met with potential residents once they had permission to approach them to explain the ISEP interview's objectives, procedures, risks and benefits. The initial informed consent discussion occurred between the researcher, the residents and the LAR if required. Written consent from residents with or without dementia or proxy consent from the LARs of those unable to give informed consent for the ISEP interview, were collected.

If a previously consenting resident declined to participate on the scheduled interview day, the interviewer tried rescheduling the interview for another day. This allowed for resolving any underlying issues or concerns that may have arisen, allowing the resident to reconsider their decision. However, if the resident declined to be interviewed for a second time, the interview will not make any further attempts to interview the resident.

The interviewer is an experienced researcher with a background in psychology (CJ) and has led the development of the ISEP tool. Due to the sensitive nature of the topics covered in the ISEP tool, the interviews were conducted individually in a tranquil environment where residents could freely discuss their preferences without privacy concerns. Interview settings included the resident's room, a quiet living space, or a secluded garden area, ensuring no other individuals were present. Since the residents were not familiar with the interviewer, sometime was allocated to allow the interviewer to establish rapport and build a comfortable relationship with them.

### 2.3 Data collection and analysis

Demographic information of residents was collected prior to the ISEP interview. During the interview, residents' responses to the questions in the ISEP tool were documented on the form. After each of the interview's conclusion, the interviewer audio-recorded her (a) experience using the ISEP tool with residents; and (b) additional insights and contextual information beyond the residents' responses to the questions in the ISEP tool. Recordings were transcribed and analyzed to inform the use of the ISEP tool and provide valuable recommendations for supporting the residents. Thematic descriptive analysis was conducted on the transcript content by a research member (CJ) and a research assistant. Differences in interpretation were discussed. The analysis focused on the areas outlined in Table 1 to assess the utilization of the ISEP tool.

**Table 1:** Content area of analysis.

**Content area**	**Description**
Acceptability	Perceived suitability of the questions in the ISEP tool • *Did residents easily understand the ISEP tool questions?* • *Was the language in the ISEP tool suitable for residents' cognitive capacity?* • *Did the questions resonate with residents in terms of importance and relevance?* • *Did the received responses align with the intended meaning of the questions?* • *Did the range of response options capture residents' diverse answers?* • *Could residents accurately recall the information needed to answer the questions?*
Usage	The extent to which the ISEP tool can be used with residents in RACFs • *Is rapport-building time required before asking sensitive questions?* • *Can sensitive questions be handled with care to reduce discomfort?* • *Were the questions in the ISEP tool logically ordered?*
Integration	The perceived fit of using the ISEP tool in RACFs • *To what extent can the ISEP be incorporated in the care process?*
Demand	Likelihood the ISEP tool will be embraced and used in RACFs • *To what extent is the ISEP tool likely to be used?*

A report with recommendations for each resident, based on their responses to the questions in the ISEP tool, was generated and provided to the facility manager with the consent of the residents. These recommendations drew upon the interviewer's expertise and the supplementary insights obtained during post-interview reflection, which went beyond the residents' responses to questions in the ISEP tool. Regular monthly teleconferences were scheduled with the facility manager and research team (i.e., interviewer) to provide follow-up on the residents' progress in supporting their sexual needs and preferences, where applicable. These meetings served as a platform to discuss and review the employed strategies to address their needs and make any necessary adjustments.

## 3 Findings

A total of 14 residents were interviewed, where there were eight female (57.14%) and six male (42.86%) residents, aged between 74 and 94 years old (M = 82.79 years, SD = 5.75). They had resided in the aged care facility between 0.15 and 4.83 years (M = 1.93 years, SD = 1.59). All residents spoke English and were neither Aboriginal nor Torres Strait Islander. Six (42.86%) of the residents had a probable dementia diagnosis. Table 2 displays frequency and descriptive statistics for the residents' demographics.

**Table 2:** Demographic variables of study residents (*n* = 14).

**Demographic variable**	***n***	**%**	***M***	***SD***	**Min**	**Max**
Age (years)			82.79	5.75	74	94
Psychogeriatric assessment scale—cognitive impairment (score)			7.64	7.09	0	21
Time since admission to residential aged care facility (years)			1.93	1.59	0.15	4.83
**Biological gender**						
Female	8	57.14				
Male	6	42.86				
**Place of birth**						
Australia	10	71.43				
United Kingdom	3	21.43				
New Zealand	1	7.14				
**First Nations Status**						
Neither Aboriginal nor Torres Strait Islander	14	100.00				
**Spoken Language**						
English	14	100.00				
**Probable diagnosis**						
Dementia	6	42.86				
Cognitive impairment	2	14.29				

### 3.1 Interviewer's (user) experience of ISEP tool

Interviews with residents lasted between 57 to 93 min (M = 74.43; SD = 9.45). When considering the areas of analysis focus (refer to Table 1) of the interviewer's experience of using the ISEP tool, the following findings are reported:

*Acceptability*—The residents generally residents generally demonstrated a good understanding of the questions in the ISEP tool, which indicates that the language used was well-suited for their cognitive capacity. Language clarity is important when working with supporting older people, including those living with dementia, as it enables them to engage meaningfully in the process. Most of the questions were well-received and elicited thoughtful and insightful responses, providing valuable information to guide care. Nonetheless, certain questions in the ISEP tool necessitated rephrasing, such as addressing barriers to expressing one's sexuality as desired and inquiring about any non-consensual sexual encounters. Additionally, further clarifications of specific terms, like the definition of “*pronoun*”, “*sexual orientation*”, and “*long-term care*” were provided to ensure that participants or residents comprehended the context and purpose of these terms. This gave more precise and nuanced insights into the residents' perspectives and needs. In general, the responses received aligned well with the intended meaning of the questions. The range of response options (e.g., Likert scale) in the tool was adequate, with most questions being open-ended. This allowed for detailed and personalized responses, offering valuable insight into their experiences and needs.

It was observed that residents with greater cognitive impairment faced challenges in maintaining focus and attention during the questioning process, where occasional repetition was necessary, accommodating their cognitive limitations. There were also instances where residents' recall of the required information seemed somewhat blurred, likely due to the effects of dementia on memory retention. This reflects the need to accommodate residents' cognitive capabilities while interpreting their responses.

*Usage—*The questions in the ISEP tool were presented in an order that facilitated a smooth and coherent assessment process. Two key themes also emerged from the interviewer's experience of using the ISEP tool:

1. Setting the stage
  *Selection of a suitable interviewer:* To conduct the ISEP interview, the interviewer should be known and trusted by the older person, such as a familiar care provider, who takes the lead. The interviewer should possess strong interpersonal skills and be capable of discussing sensitive information without causing unnecessary discomfort or distress.
  *Selection of a suitable environment:* The discussion should be in a quiet and private area to provide the older person with the necessary confidentiality. Their bedroom or a secluded area in the garden can be suitable settings. It is important to be mindful of others present, ensuring the conversation is not overheard. If the interview occurs in a communal space, awareness of passersby and maintaining an appropriate vocal volume are important to ensure the older person's privacy.
2. Initiation of the ISEP interview
  Building rapport and establishing trust is essential, especially if the interviewer is unfamiliar with the older person. The interviewer needs to self-introduce, explain their presence and role, and learn about the older person, including asking how they prefer to be addressed. A clear explanation and the purpose of the ISEP tool should be provided to the older person to assure them that their answers are confidential and that they cannot answer any uncomfortable questions. The interview process needs to be briefly outlined, including the types of questions and response options. To ensure the comfort and privacy of the older person during the discussion of personal information in the ISEP tool, approach the conversation with sensitivity and respect. The older person should guide the discussion, allowing them to set the pace and topics to be addressed. Maintaining a comfortable atmosphere includes making light of the discussion to ease tension or discomfort and addressing any distress the older person may express.

Moreover, concerning *Demand* and *Integration*, the post-ISEP tool usage discussion with the facility manager highlighted a significant opportunity to incorporate the ISEP as part of the standard practice. This is due to the increasing reports of behaviors related to intimacy and sexuality among residents. By utilizing the ISEP, the facility can better understand the needs and preferences of the residents, enhancing the overall care provided to them.

### 3.2 Individual report with recommendations

The individual reports with recommendations generated for each resident after analyzing their responses to the questions in the ISEP tool were very important to inform the discussion between the facility manager and the research team (i.e., interviewer). It allows for facilitated discussions on strategies to address the residents' needs and make any necessary adjustments. These meetings served as a valuable avenue to review and refine the support provided.

*Resident example: Mabel*
  Before the ISEP interview with Mabel, her daughter shared concerns regarding her close relationship with another male resident. It was noted that Mabel was frequently found lying in his bed and occasionally in the beds of other residents, regardless of their gender. Although the male resident with mid-stage dementia seemed indifferent to her presence, his visiting wife was not appreciative of Mabel's actions. Additionally, other residents affected by her behaviors found them upsetting and some reacted with anger. This information set the context for the interview.
  During the interview, Mabel abruptly stood up a few minutes in, walked over to the male resident's room, used his bathroom, and was later found lying in his bed. Care staff intervened and removed her from his room. Throughout the interview, Mabel continued to wander and attempted to lie on other residents' beds, a behavior that staff identified as typical for her. Despite these interruptions, Mabel could provide short and clear responses during the interview. However, due to her cognitive impairment, she struggled with questions that required explanation or elaboration, such as understanding the concept of intimacy. Multiple attempts to understand the reasons behind Mabel's behaviors made it apparent that she may not seek romantic or sexual relationships but companionship and company due to feelings of loneliness. This explanation helped make sense of her behaviors of entering other residents' rooms, lying on their beds, and seeking physical closeness. Based on these observations and insights, the following recommendations were proposed:

a) Monitoring Mabel's behavior while acknowledging that her actions may be driven by a desire for intimacy and companionship rather than romantic or sexual motives.
b) Purchase of a cuddle comfort pillow as a potential intervention. This pillow may help alleviate Mabel's feelings of loneliness and reduce her tendency to enter other residents' rooms, lie on their beds, and seek physical closeness.

In follow-up meetings with the facility manager, the effectiveness of providing the cuddle comfort pillow for Mabel was discussed. Initially, the pillow positively addressed her behavior by providing companionship and/or comfort. However, as her dementia progressed, her behavior briefly resumed before ceasing.

## 4 Discussion and conclusion

This paper reports on the use of the ISEP tool among long-term aged care residents, focusing on individuals with dementia, through a case study centered on user experience. To our knowledge, the ISEP tool stands as the sole known instrument explicitly crafted to facilitate discussions concerning the intimacy and sexuality preferences of older adults, particularly those in long-term care settings. Consequently, there are no direct alternatives or comparable tools with equivalent objectives available for comparison. While there do exist assessment tools that measure relationship intimacy (i.e., Personal Assessment of Intimacy in Relationships) (Schaefer and Olson, 1981) and assess the sexuality of older adults (i.e., PLISSIT model) (Annon, 1976), as well as tools designed to review policy development aimed at supporting and optimizing the expression of sexuality in residential aged care facilities (i.e., Sexuality Assessment Tool) (Bauer et al., 2014), it is essential to note that these tools serve different purposes and focus areas. Additionally, there are tools that measure specific areas of male or female sexual functioning and satisfaction (Golombok-Rust Inventory of Sexual Satisfaction for Males/Females) (Rust and Golombok, 1985), but these also serve distinct purposes.

Overall, findings from the interviewer's post-reflection indicated that residents demonstrated a general understanding of the questions in the ISEP tool, which appeared appropriate for their cognitive capacity. Not only is it important to establish a rapport with residents beforehand, but it is also crucial to exercise sensitivity and care when asking the ISEP tool questions. However, residents with advanced dementia faced challenges in maintaining focus, leading to the need for occasional repetition or rephrasing of questions. The response options, mostly open-ended, were sufficient, although some questions required additional clarification to elicit meaningful responses. Overall, residents' answers aligned with the intended meaning of the questions, despite occasional difficulties with recall. Selection of suitable interviewers, fostering a conducive environment, and allowing ample time for rapport-building were important factors leading to open disclosure of needs and preferences. Outcomes of the case study suggest that the ISEP tool can be used in RACFs, including with older adults and those with dementia.

The summary of responses obtained through the ISEP tool presented as a report, offers a practical overview of key discussion points and recommendations. It is a foundation for engaging in constructive dialogue with the facility manager. It facilitates the consideration, employment, and evaluation of practical and actionable strategies. These strategies aim to address the identified challenges and enhance the current situation. This documentation aligns with established aged care practices, as it captures interview details, promotes communication among the healthcare team, ensures continuity of care, assists in care planning, and enables evaluation and monitoring of the older person's progress (Keenan et al., 2008; Aged Care Quality and Safety Commission, 2012). The presented resident example of Mabel illustrates how her longing for companionship can be met respectfully, emphasizing her wellbeing and creating a nurturing atmosphere within the RACF. By examining and assessing the implemented strategies, identified needs and preferences of Mabel can be effectively addressed and evaluated. The report provides aged care providers with valuable insights to initiate conversations that can potentially pave the way for positive change and help achieve desired outcomes by adopting a person-centered care approach.

It should be acknowledged that although the study demonstrated the potential of using the ISEP tool in aged care facilities to enhance care practices to address the intimacy and sexuality needs of older people with and without dementia, it is important to consider that the reported findings were based on the case study of a single aged care facility with a small group of residents. It is acknowledged that the credibility of both the case study and its findings may be potentially influenced by the sole interviewer, who also happens to be the developer of the ISEP tool and one of the two individuals involved in data analysis. Therefore, conducting further large-scale studies with multiple facilities, including those that are non-heteronormative or gender-diverse identity-friendly, is necessary to validate the external applicability of the ISEP tool on a broader scale. This will provide more robust evidence and ensure that the benefits of the ISEP tool can be effectively realized across diverse aged care settings.

Furthermore, the absence of a user guide for the ISEP tool currently hinders its potential utilization and should be the next step in advancing work on the ISEP tool. The findings from this study hold the promise of informing the creation of a comprehensive user guide, one that goes beyond mere instruction and acts as an important manual for aged care providers seeking to use the ISEP tool. This guide can extend its support by offering essential insights into reporting and interpreting the findings, guiding the identification of appropriate actions, aiding in informed decision-making, and facilitating outcome evaluation. Accommodation for residents' cognitive states both in interpreting their responses and in formulating recommendations from ISEP results should also be clearly outlined in the proposed user guide. To enhance accessibility and knowledge dissemination, a dedicated website can be established as a centralized resource hub. This platform would encompass the ISEP tool itself, the practical user guide, illustrative case studies, supplementary resources, and reporting templates. Such an inclusive approach can potentially enrich the understanding and implementation of the ISEP tool in aged care settings, fostering person-centered dementia care that accommodates the intimacy and sexuality needs, preferences, and expressions of all individuals involved.

## Data availability statement

The datasets presented in this article are not readily available because of the sensitivity of the qualitative data. Requests to access the datasets should be directed to cjones@bond.edu.au.

## Ethics statement

The studies involving humans were approved by Bond University Human Ethics and Research Committee. The studies were conducted in accordance with the local legislation and institutional requirements. Written informed consent for participation in this study was provided by the participants' legal guardians/next of kin.

## Author contributions

CJ: Writing – original draft, Writing – review & editing. WM: Writing – original draft, Writing – review & editing. KV: Writing – original draft, Writing – review & editing. CH: Writing – original draft, Writing – review & editing.
